# Boolean Models of Genomic Regulatory Networks: Reduction Mappings, Inference, and External Control

**DOI:** 10.2174/138920209789177584

**Published:** 2009-09

**Authors:** Ivan Ivanov

**Affiliations:** Veterinary Physiology and Pharmacology, Texas A&M University, College Station, TX 77843-4466, USA

## Abstract

Computational modeling of genomic regulation has become an important focus of systems biology and genomic signal processing for the past several years. It holds the promise to uncover both the structure and dynamical properties of the complex gene, protein or metabolic networks responsible for the cell functioning in various contexts and regimes. This, in turn, will lead to the development of optimal intervention strategies for prevention and control of disease. At the same time, constructing such computational models faces several challenges. High complexity is one of the major impediments for the practical applications of the models. Thus, reducing the size/complexity of a model becomes a critical issue in problems such as model selection, construction of tractable subnetwork models, and control of its dynamical behavior. We focus on the reduction problem in the context of two specific models of genomic regulation: Boolean networks with perturbation (*BN_P_*) and probabilistic Boolean networks (*PBN*). We also compare and draw a parallel between the reduction problem and two other important problems of computational modeling of genomic networks: the problem of network inference and the problem of designing external control policies for intervention/altering the dynamics of the model.

## 1. INTRODUCTION

One can think of a *Gene Regulatory Network* (GRN) as a network of relations among strands of DNA (genes) and the regulatory activities associated with those genes [1]. This general definition allows for many mathematical (usually dynamical) systems to be called GRNs. The goodness of such models is evaluated using several important criteria: the level of description of the biochemical reactions involved, the complexity of the model, the model parameter estimation, and its predictive power. There have been many attempts to model the structure and dynamical behavior of GRNs, ranging from deterministic with discrete time space to fully stochastic with continuous time space [2]. The well known central 'dogma' of molecular biology implies that genes communicate *via *the proteins they encode [3]. Both stages of protein production, transcription and translation, are controlled by a multitude of biochemical reactions, and are influenced by both internal and external to the cell factors. This perspective suggests that the expression of a given gene *i* , i.e. the quantity of either protein or messenger RNA, should be considered as a random function $X_{i}\left(t\right)$ of the cell's internal and external environments. Thus, if one wants to study the dynamical behavior of a GRN, one must design a mathematical model for the gene-expression vector $X\left(t\right)=\left(X_{1}\left(t\right),X_{2}\left(t\right),...,X_{n}\left(t\right)\right)$ for the n genes that form the network. The stochastic differential equation model appears to provide the most detailed description of the dynamics of **X**(*t*) . In principle, it could include all of the information about the biochemical processes involved in gene regulation. At the same time, the estimation of its parameters cannot be done without large amount of reliable time-series data [4]. A more pragmatic approach is to look for simpler models for the dynamics of the gene-expression vector. One of the most extreme simplifications is the Boolean network model, originally proposed by Kauffman [5]. The Boolean network model is based on the observation that during the regulation of its functional states the cell often exhibits switch-like behavior. Recent work using the NCI 60 anti-cancer drug screen has demonstrated that Boolean logic type interactions can be detected in gene expression data [6]. While there are instances in gene regulation where the Boolean logic is the appropriate level of description of the interactions - for instance, when transcription factors have to form a complex that binds to the cis-regulatory DNA to activate transcription, one should keep in mind that discrete models cannot capture the details of the biochemical reactions involved in those processes. However, it is not the binary nature of the Boolean network model that is its greatest weakness, one even more important deficiency is its determinism. Deterministic models, such as the Boolean network, cannot represent the consequential perturbations due to external latent variables. In addition, the Boolean network model in its original formulation cannot be used to represent biologically meaningful events, such as gene mutations. Its stochastic extension - probabilistic Boolean network (PBN), was introduced by Shmulevich *et al*. in an attempt to account for those latent variables and gene perturbations while keeping the Boolean logic as the model for the gene-gene interactions [7, 8]. The PBN model is an example of a well studied discrete stochastic dynamical system, and has been successfully applied in situations where data come from cells operating in different contexts or include noisy observations, which implies that the model has to account for that randomness. The dynamics of a PBN can be studied in the context of Markov chains which allows for the development of control theory for the purposes of intervention. Being a collection of Boolean networks with a probability structure, the PBN model could be viewed as a minimal extension of the Boolean network which allows for modeling of the stochastic nature of complex systems with latent variables and random experimental effects. However, even such a minimal extension of the deterministic model exhibits high complexity which impedes its practical applications to model GRN of more than 40 genes. Hence, there is a need for constructing size-reducing mappings that produce new and more tractable models that share some of the biologically meaningful properties of the larger-scale models.

## 2. DEFINITIONS OF *BN_P_* AND *PBN*. INFERENCE FROM DATA AND EXTERNAL CONTROL

The initial application of Boolean networks as a model of genomic regulation was to study the evolution of ensembles of networks which were restricted to a specific type of fitness landscape [5, 9]. Here we provide the definition of a Boolean network and briefly discuss the ensemble approach.

A Boolean network $\mathrm{BN}=\left(V,f\right)$ on *n* genes is defined by a set of nodes/genes $V=\left\{x_{1},...,x_{n}\right\}$ and a vector of Boolean functions $f=\left[f^{1},...,f^{n}\right]$.

Each variable $x_{i}\in \left\{0,1\right\}$ represents the expression level of the respective gene *i* , with 1 representing high and 0 representing low expression. The vector **f** represents the regulatory rules between genes. At every time step *t* + 1, the value of *x_i_* is predicted by the values of a set *W_i_* of genes at the previous time step *t* , based on the regulatory function$f^{i},\;i.e.\;\;x_{i}\left(t+1\right)=f^{i}\left(x_{i_{1}}\left(t\right),...,x_{i_{k_{i}}}\left(t\right)\right).$ The set of genes $W_{i}=\left\{x_{i_{1}},...,x_{i_{k_{i}}}\right\}$ is called the predictor set of *x_i_*, and the function $f^{i}$ is called the predictor function of *x_i_*. The pairs $\left(x_{i},W_{i}\right),i=1,...,n$ induce a digraph *G* with edges $x_{i_{j}}\to x_{i}$ representing the structural dependencies among the genes. A state of *BN* is a vector $s=\left[x_{1},...,x_{n}\right]\in {\left\{0,1\right\}}^{n}$. All of the states of the Boolean network *BN* comprise its state space *S* which combined with the functions in **f** produces a digraph Γ called the state transition diagram of *BN*. Γrepresents the dynamics of the Boolean network and can be identified with a $2^{n}\times 2^{n}$ matrix *P* with rows and columns indexed by the states in *BN* and entries $p_{\mathrm{ij}}=p\left({\text{s}}_{i},{\text{s}}_{j}\right)=1$ if there is a transition from the state ${\text{s}}_{i}\to {\text{s}}_{j}$ in *S* or 0 otherwise. Given an initial state, the network will eventually enter a set of states in *G* through which it will repeatedly cycle forever. Each such set is called an attractor cycle, and a singleton attractor is an attractor cycle of length 1. The network attractors induce a partition of state space *S* where the subset of states that belong to the same equivalence class is called the basin of the corresponding attractor cycle. The attractors of a Boolean network represent a type of memory of the dynamical system [10].

Originally, [11], analytical results and numerical simulations based on ensembles of randomly generated Boolean nets focused on the relationships between the structural gene interdependencies and dynamical behavior of the ensembles. Those studies provided insights into the general characteristics of large GRNs and the related evolutionary principles. 'Tuning up' of ensemble parameters such as the average connectivity *K* and the predictor functions' bias *p* can be used to study the operating regimes of the networks. The average connectivity is defined as the average size of the predictor sets $W_{i},$ and the bias *p* is defined as the probability of a given predictor function to assume a value of 1. Depending on the values of *K* and *p* there are two main modes of operation of a *BN* : ordered and chaotic. In the ordered regime most of the system components/nodes are frozen at either 1 or 0 value, and the transfer of information is impeded by those large frozen islands of genes. In the chaotic regime, the system is very sensitive to small perturbations where a change of the value of one node can propagate to many others in an avalanche-like manner. The phase transition boundary between the ordered and the chaotic regimes is called the complex regime or critical phase. It has been shown that Boolean networks in that regime are the most evolvable and Kauffman [11] argues that life must exist on that edge between order and chaos: "a living system must first strike an internal compromise between malleability and stability. To survive in a variable environment, it must be stable to be sure, but not so stable that it remains forever static". Structural stability is one of the central concepts in the theory of dynamical systems. It describes persistent behavior that cannot be destroyed by small changes to the system. As real GRNs are capable of maintaining metabolic homeostasis and stable developmental program in the face of a changing environment, they certainly possess structural stability. The Boolean network model naturally captures this phenomenon because the network 'flows' back to one of its attractors after a small gene perturbation. Following this line of reasoning, Kauffman [11] suggests that the attractors in a *BN* correspond to cellular types. Another interpretation of the attractors of a *BN* is that they represent cellular states, such as proliferation (cell cycle), apoptosis (programmed cell death), and differentiation (execution of cell-specific tasks) [12, 13]. For example, if a structural perturbation (mutation) happens which moves the network from the basin of the apoptotic attractor, the cells could exhibit uncontrolled growth or hyper proliferation, typical of tumorigenisis. The two interpretations of the attractors in the Boolean network model are complementary to each other: for a given cell type, different functional states exist and are determined by the collective gene activity. Thus, a particular cell type can encompass several attractor cycles each one corresponding to different cellular functional states. We refer the reader to [11, 14, 15] for a detailed treatment and additional references to results about the interplay between the average connectivity and the bias of the predictor functions in a *BN* and how that impacts the dynamical behavior of the network. An important implication from the body of work on the effects of these local parameters on the network is that if one wants to model GRNs with Boolean networks or their generalizations one should constraint the network connectivity in order to keep the model on the edge of chaos and closer to the ordered regime. For example, in the case of unbiased, *p* = 0.5, predictor functions the networks with K>2 operate mostly in the chaotic regime which renders such models incompatible with the real GRNs which are clearly non-chaotic systems. Although the ensemble studies can provide important insights into some general properties of the Boolean network models, a single Boolean network itself is not capable of capturing the effects of latent variables or random gene perturbations. Moreover, the ensemble approach does not provide a way of explicitly inferring the specific *BN* structure from data, e.g. cDNA microarray gene expression. Inferring the *BN* structure from data has the potential to reveal how to design therapeutic intervention for GRNs which show a specific disease phenotype. It should be pointed out that the data used for network inference exhibits uncertainty on various levels. First, due to biological variability, gene expression is inherently stochastic. Second, the complex measurement process, the microarray preparation, image acquisition and processing create experimental noise that has to be taken into account during the inference of the network. All of this combined with the presence of latent or unobservable variables such as proteins or environmental conditions present us with the problem to infer deterministic predictor functions under uncertainty. To solve such a problem one needs to reliably estimate the uncertainty. Without such estimation one cannot be sure how the designed predictor function will perform when presented with new data.

One possible way to approach this problem was proposed by Shmulevich *et al*. [7], and Brun *et al*. [8]. Keeping in mind that the predictor functions cannot be reliably estimated from the limited amount of data relative to the number of genes on a microarray slide, one can infer a number of simple predictor functions, each of which performs relatively well in predicting the target gene. Here, simpler is understood as having predictor sets $W_{i}$ of smaller size. After producing such predictor functions, one has to combine them together accounting for the uncertainty at the same time. This 'probabilistic' approach to synthesize 'good' predictor functions leads to the PBN model of genomic regulatory networks.

A binary PBN $A=A^{q,p,r}\left(V,F,C\right)$ is defined by a set of nodes/genes $V=\left\{x_{1},...,x_{n}\right\},$ a set of vector-valued Boolean functions $\text{F}=\left\{{\text{f}}_{1},...,{\text{f}}_{r}\right\},{\text{f}}_{j}:{\left\{0,1\right\}}^{n}\to {\left\{0,1\right\}}^{n},j=1,...,r$ called realizations of *A* or network functions, a list of selection probabilities $C=\left\{c_{1},...,c_{r}\right\}$ for the corresponding realizations, a gene mutation/flipping probability $p\in \left[0,1\right],$ and a realization switching probability $q\in \left[0,1\right],$.

The original definition of the PBN model [7] concerned the instantaneously random PBN model only, i.e. the model where *p* = 0 and *q* = 1. When the parameters *p* > 0 and *q* < 1 the PBN is said to be *context-sensitive* [8]. The context-sensitive PBNs allow for the interpretation of data obtained from distinct sources, each representing a specific cell context. Thus, one interprets data as obtained from a family of deterministic *BN* , and the PBN is viewed as a collection of Boolean networks in which one constituent network governs the gene activity for a random period of time before another randomly selected deterministic *BN* takes over which might be in response to external stimulate or activity of latent variables.

Updating the values of all genes in the network at time *t* is done synchronously according to the components of the currently used network function, and then the process is repeated. The choice of which network function ${\text{f}}_{j}$ to apply is governed by a selection procedure. Specifically, at each time point *t* a random decision is made as to whether to switch the network function for the next transition, with a probability *q* of a switch being a system parameter. If a decision is made to switch the network function, then a new realization is chosen from among all of the possible realizations of ${\text{f}}_{j}\in \text{F}$ of *A* , according to their individual selection probabilities $c_{j}\in C$. In other words, each network function ${\text{f}}_{j}$ represents a deterministic *BN* and the PBN behaves as a fixed *BN* until a random decision (with probability of *q*) is made to change the network function according to the probabilities $\left\{c_{1},...,c_{r}\right\}$ from among $\left\{{\text{f}}_{1},...,{\text{f}}_{r}\right\}$ . Notice that the co-ordinates $f_{j}^{i},i=1,...,n$ of each realization ${\text{f}}_{j}$ are the predictor functions of the Boolean network determined by that network function.

In addition to the network switching and selection in the PBN model, there is mechanism which models random gene mutations, i.e. at each time point *t* there is a probability *p* of any gene changing its value uniformly randomly. Thus, the PBN model can account for the uncertainties in both data and model selection. The PBN *A* shares the same state space *S* with its realizations, and the state transition diagrams Γ_j_ of the individual $B_{j}$'s combine naturally into a stochastic state transition diagram Γ representing the dynamics of *A* . As in the case of deterministic Boolean networks, Γ can be identified with a stochastic $2^{n}\times 2^{n}$ matrix *P* , also known as transition probability matrix, with non-negative entries $p_{\mathrm{ij}}$ and having the property $\sum_{j=1}^{n}p_{\mathrm{ij}}=1,i=1,...,n$. Using this matrix, the dynamics of *A* can be described using the well-developed theory of Markov chains. One should notice that if the probability of gene flipping *p* is positive then the Markov chain representing the dynamics of the network is ergodic which implies that it possesses a *steady-state probability distribution π*

The synchronicity requirement for the state transitions in a PBN is an oversimplification of the real interactions that take place during genomic regulation. While it is not difficult to extend the PBN model into an asynchronous one [16], we do not discuss such extensions here. There are two reasons for focusing our attention to the synchronous case of PBN only. First, the model estimation from data is a much harder problem for asynchronous compare to the case of synchronous PBNs. Second, the synchronous PBN framework facilitates a simpler and clearer treatment of the problems about complexity-reducing mappings.

One can also view a given context-sensitive PBN *A* as a collection of *r* Boolean networks with perturbation ${\mathrm{BN}}_{p}^{j},j=1,...,r$. These building blocks of *A* are obtained by adjoining the gene perturbation probability *p* to each one of the deterministic Boolean networks that represent the *r* possible contexts of *A* . Thus

A Boolean network with a perturbation $p,{\mathrm{BN}}_{p}=\left(V,\text{f}\right),$ on *n* genes is defined by a set of nodes $V=\left\{x_{1},...,x_{n}\right\},$ a vector of Boolean functions $\text{f}=\left[f^{1},...,f^{n}\right],$ and the gene mutation/flipping probability $p\in \left[0,1\right]$.

It is obvious that a Boolean network with a perturbation is a special case of a PBN with just one context, *r* =1. Just as in the general case of a PBN, the dynamics of a ${\mathrm{BN}}_{P}$ is represented by a Markov chain. The Markov chain of a ${\mathrm{BN}}_{P}$ is completely described by its transition probability matrix $P={\left(p\left({\text{s}}_{i},{\text{s}}_{j}\right)\right)}_{i,j=1}^{2n},$ where $p\left({\text{s}}_{i},{\text{s}}_{j}\right)$ is the probability of the chain undergoing the transition from the state ${\text{s}}_{i}$ to the state ${\text{s}}_{j}$. The perturbation probability *p* makes the chain ergodic, and thus, it possesses steady-state probability distribution. Computing the elements of *P* is straightforward and we elect to present it here because of its importance in the subsequent considerations. When computing the transition probabilities for a ${\mathrm{BN}}_{P}$ one has to realize that at every time step one of the two mutually exclusive events happens: either the chain transitions according to the regulatory rules **f** or a perturbation occurs. This interpretation implies that when no perturbation occurs the network regulatory rules are applied. There are two important cases in computing $p\left(\text{s},{\text{s}}_{j}\right)$ for every given state s∈S. The first case is when **s** is a singleton attractor, i.e. **f (s) = s** . In that case one can easily see that $p\left(s,s_{j}\right)=p^{k_{j}}{\left(1-p\right)}^{n-k_{j}},$, where $k_{j}$ is the number of the positions where the binary representations of **S** and ${\text{s}}_{j}$ differ from each other, i.e. the Hamming distance between the two states. The second case is when **f(s) = s*_i_***, where ${\text{s}}_{i}\neq \text{s}$ . Clearly, in this case,$p\left(\text{s},\text{s}\right)=0,$ and $p\left(\text{s},{\text{s}}_{j}\right)=p^{k_{j}}{\left(1-p\right)}^{n-k_{j}},$ , for ${\text{s}}_{j}\neq {\text{s}}_{i}$. The transition $\text{s}\to {\text{s}}_{i}$ can happen by either applying the regulatory rules **f** with a probability of ${\left(1-p\right)}^{n}$ or by perturbation with a probability of $p^{k_{i}}{\left(1-p\right)}^{n-k_{i}}.$. Thus, $p\left(s,s_{i}\right)={\left(1-p\right)}^{n}+p^{k_{i}}{\left(1-p\right)}^{n-k_{i}}.$ .

The interpretation of a PBN as a collection of *r* Boolean networks with a perturbation allows to view the Markov chain representing the dynamics of the PBN as a collection of *r* Markov chains with a switching mechanism between them. The switching rules are defined by the PBN switching probability *q* and the set of selection probabilities *C* . This kind of interpretation of the dynamics of a PBN is advantageous when one considers problems related to control and reduction of the model's complexity.

One of the main objectives of developing mathematical models of genomic regulation is the identification of potential targets for therapeutic intervention [10]. For example, the abundance of mRNA for the gene WINT5A has been shown to discriminate well between cells' low or high metastatic competence [17]. This suggests that altering the expression of WINT5A could be perceived as a goal of a possible therapeutic intervention [18]. The PBN model of genomic regulation provides an appropriate setting for studying optimal regulatory intervention. The question about control and intervention can be posed in terms of the dynamics of the underlying Markov chain [7]. There are two different types of effects of external/control variables on the dynamical evolution of the network: either the finite-time or the infinite-time ones. The short-term control policies have been shown to affect the dynamics of the model over a small number of stages; however they do not always achieve the desired change in the long-run network behavior [19, 20]. The infinite-horizon intervention strategies have been studied using stochastic control combined with dynamic programming algorithms. This approach has led to finding of stationary control policies that affect the steady-state distribution of a given PBN? Another important problem in the study of the infinite-horizon control is the identification of the best intervening gene.

The direct approach of solving the optimal control problem for each gene in the model and comparing the performance of the respective control policies is a computationally expensive procedure because the complexity of the dynamic programming algorithms increases exponentially with *n* -the number of genes [22]. Thus, there is a need to develop less complex algorithms for designing of sub-optimal intervention policies. Vahedi *et al*. [23] used a biology motivated approach to find such policies based on the *mean first-passage times* (MFPT) of the states s∈S [10, 24]. Instead of formulating the problem about designing the optimal control in its full generality we elect to discuss a simpler version of the MFPT control policy algorithm based on a single control gene *g* because it facilitates the analysis of the interplay between reduction mappings and control policies for the PBN model. A control policy $\pi_{g={\left\{\mu_{g}\left(t\right)\right\}}_{t>0}}$ is defined as a sequence of decision rules $\mu_{g}\left(t\right):S\to \left\{0,1\right\}$ for each time step *t* , [21]. The values 0/1 are interpreted as off/on for the application of the control. The MFPT algorithm is based on the comparison between the MFPT of a state **s** and its *flipped* with respect to *g* state ${\tilde{\text{s}}}^{g}$, i.e. the state that differs from **s** only in the value of *g* . When considering therapeutic interventions the state space *S* can be partitioned into desirable *D* and undesirable *U* states according to the expression values of a given set *W* of genes. For simplicity we will assume that *W* = {*x*}. The intuition behind the MFPT algorithm is that given the control gene *g* , when a desirable state **s** reaches *U* on average faster than ${\tilde{\text{s}}}^{g}$, it is reasonable to apply control and start the next network transition from ${\tilde{\text{s}}}^{g}$ . The roles of **s** and ${\tilde{\text{s}}}^{g}$ are reversed when s∈U . Without loss of generality one can assume that the gene *x* is the leftmost gene in the states' binary representations, i.e. $x_{1}=x$ and $\text{s}=\left[x,x_{2},...,x_{n}\right]$ and the desirable states correspond to the value x=0. With this assumption, the probability transition matrix *P* of the Markov chain representing the PBN can be written as

(1) $$P=\left(\begin{array}{c} \begin{array}{cc} P_{\mathrm{DD}} & P_{\mathrm{DU}} \end{array}\\ \begin{array}{cc} P_{\mathrm{UD}} & P_{\mathrm{UU}} \end{array} \end{array}\right)$$

Using this representation one can compute the mean first-passage times *K_U_* and *K_D_* by solving the following system of linear equations [25].

(2) $$K_{U}=e+P_{\mathrm{DD}}K_{U}$$

(3) $$K_{D}=e+P_{\mathrm{UU}}K_{D}$$

where *e* are unit vectors of the appropriate length. The vectors *K_U_* and *K_D_* contain the MFPTs from each state in *D* to the set *U* , and from each state in *U* to the set *D* respectively. The MFPT algorithm designs stationary control policies $\pi_{g,\gamma }=\left\{\mu_{g,\gamma },\mu_{g,\gamma },...\right\}$ for each gene *g* in the network by comparing the differences $K_{D}\left(\text{s}\right)-K_{D}\left({\tilde{\text{s}}}^{g}\right)$ and $K_{U}\left({\tilde{\text{s}}}^{g}\right)-K_{U}\left(\text{s}\right)$ to *γ* - a tuning parameter. The parameter *γ* is set to a higher value when the ratio of the cost of control to the cost of the undesirable states is higher, the intent being to apply the control less frequently. It is important to notice that while the MFPT control policy $\pi_{g,\gamma }$ is a sub-optimal one it can approximate well the optimal control policy which being a solution to the Bellman optimality equation is also a stationary one [23, 26, 27].

## 3. REDUCTION MAPPINGS FOR *PBN*

High complexity, both model-wise and computational, is a major impediment for the practical applications of mathematical models of genomic regulation. Hence, there is a need for size reducing mappings producing new and more tractable models that share some, preferably all, of the biologically meaningful properties of the larger-scale models. The most common approach for reducing the complexity of a network model of genomic regulation is by 'deleting' a gene from the model. One of the first such mappings, the *projection mapping*, was proposed in ? as an attempt to reduce the complexity of an independent instantaneously random PBN *A* while maintaining consistency with its original probability structure. Here, following [28], we provide the definition of the projection mapping for the general case of a context-sensitive PBN. The basic projection $\Pi_{i}$ is a mapping that transforms the given PBN *A* into a new one with the same parameters *q* and *p* , and such that the number of genes is reduced by one, i.e. the gene *x_i_* in the original network is 'deleted'. Without loss of generality one may assume that the deleted gene is *x_n_*. Thus, for a PBN *A*

${\hat{\Pi }}_{n}:A\to {\hat{A}}_{n}$

${\hat{A}}_{n}^{q,p,s}\left(\hat{V},\hat{F},\hat{C}\right),\hat{V}=\left\{x_{1},...,x_{n-1}\right\},$

$\hat{F}=\left\{{\hat{\text{f}}}_{1}{\hat{\text{f}}}_{2},...,{\hat{\text{f}}}_{2}\right\},\;\hat{C}=\left\{{\hat{c}}_{1},...,{\hat{c}}_{s}\right\}$

Every predictor function $f_{j}^{\left(i\right)},i=1,...,n-1,$, generates two predictors ${\hat{f}}_{0j}^{\left(i\right)}$ and ${\hat{f}}_{1j}^{\left(i\right)}$ according to the rule

(4) $${\hat{f}}_{\mathrm{kj}}^{\left(i\right)}\left(x_{1},..,x_{n-1}\right)=f_{j}^{\left(i\right)}\left(x_{1},..,x_{n-1},k\right)$$

$k\in \left\{0,1\right\},\forall \left(x_{1},..,x_{n-1}\right)$ in ${\hat{A}}_{n}$

Thus, every network function ${\text{f}}_{j}$ for *A* determines $2^{n-1}$ new network functions for ${\hat{A}}_{n}$ by combining ${\hat{f}}_{0j}^{\left(i\right)}$'s and ${\hat{f}}_{1j}^{\left(i\right)}$'s in all of the possible ways for every fixed *j* and i=1,...,n−1 . The new network functions have their corresponding selection probabilities given by the formula

(5) $$c_{j}{\left(\mathrm{Pr}\left\{x_{n}=1\right\}\right)}^{l}{\left(\mathrm{Pr}\left\{x_{n}=0\right\}\right)}^{n-l-1},\;j=1,...,r$$

where *l* is the number of the components of the new network function that are coming as ${\hat{f}}_{1j}^{\left(i\right)}$, and $P_{r}\left\{x_{n}=k\right\},k\in \left\{0,1\right\}$ is the marginal probability for the gene $x_{n}$ to have values 0 or 1, computed using the steady/stationary state probability distribution of the original PBN. For example, the new network function $\left({\hat{f}}_{11}^{\left(1\right)},{\hat{f}}_{01}^{\left(2\right)},...,{\hat{f}}_{01}^{\left(n-1\right)}\right)$ has its selection probability equal to $c_{1}\left(\mathrm{Pr}\left\{x_{n}=1\right\}\right){\left(\mathrm{Pr}\left\{x_{n}=0\right\}\right)}^{n-2}$ . When two or more of the network functions for ${\hat{A}}_{n}$ happen to be identical their selection probabilities combine in a natural way. This mapping preserves the probability structure of a PBN but the number of the BNs that compose the resulting PBN could be exponentially larger compare to the number of the BNs forming the original PBN. Thus, the projection mapping can not be used to reduce the complexity of a PBN model of genomic regulation. Moreover, it shows that in general, a reduction mapping could be a one-to-many mapping with the potential to increase the complexity of the network model.

A different kind of size-reducing mapping (which also preserves the parameters *q* and *p* of the original PBN) is the *reduction mapping*, see [29] for the special case of an independent instantaneously random PBN. It is important to point out that this mapping might not preserve the probability structure of the original PBN. Instead, it aims at reducing the model's complexity. To better understand the motivation and the definition of the reduction mapping we consider the following portion of the probabilistic state transition diagram Γ for the original PBN containing the states ${\text{s}}_{1}=\left[x_{1},...,x_{n-1},1\right],{\text{s}}_{0}=\left[x_{1},...,x_{n-1},0\right],{\text{v}}_{1}=\left[{x^{\prime }}_{1},...,{x^{\prime }}_{n-1},1\right],$ and ${\text{v}}_{0}=\left[{x^{\prime }}_{1},...,{x^{\prime }}_{n-1},0\right]$


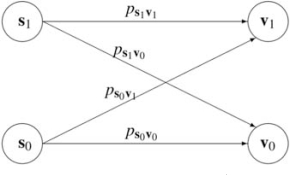


where $p_{s_{1}v_{1}},p_{s_{0}v_{0}},p_{s_{1}v_{0}},p_{s_{0}v_{1}}$ are the corresponding transition probabilities. If one 'deletes' the node *x_n_* this diagram collapses to


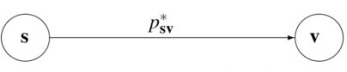


 where $\text{s}=\left[x_{1},..,x_{n-1}\right]$ and $\text{v}=\left[{x^{\prime }}_{1},..,{x^{\prime }}_{n-1}\right]$ are the corresponding states, and $p_{\mathrm{sv}}^{\text{∗}}=\mathrm{Pr}\left\{x_{n}=1\right\}\left(p_{s_{1}v_{1}}+p_{s_{1}v_{0}}\right)+\mathrm{Pr}\left\{x_{n}=0\right\}\left(p_{s_{0}v_{1}}+p_{s_{0}v_{0}}\right)$

If we formally define the reduction mapping acting on a PBN $A^{q,p,r}\left(V,F,C\right)$ by 'deleting' one gene as:

${\tilde{\Pi }}_{n}:A\to {\tilde{A}}_{n}$

${\tilde{A}}_{n}^{q,p,s}\left(\tilde{V},\tilde{F},\tilde{C}\right),\tilde{V}=\left\{x_{1},...,x_{n-1}\right\}$

$\tilde{F}=\left\{{\tilde{\text{f}}}_{1},{\tilde{\text{f}}}_{2},...,{\tilde{\text{f}}}_{s}\right\},\tilde{C}=\left\{{\tilde{c}}_{1},...,{\tilde{c}}_{s}\right\}$

and require that s≤r for the new PBN ${\tilde{A}}_{n}$ with network functions having the same selection probabilities as their counterparts in $A,{\tilde{c}}_{j}=c_{j},j=1,...,s,$ we can see that in order to maximally preserve the probability structure of the original PBN, the probabilistic state transition diagram for ${\tilde{A}}_{n}$ must have transition probabilities closely matching the transition probabilities $p_{\text{sv}}^{\text{*}}$ of the 'collapsed' state transition diagram described above. This goal is achieved by an optimization procedure which for every fixed network function ${\text{f}}_{j},j=1,...,s$ from *A* combines the predictor functions ${\hat{f}}_{0j}^{\left(i\right)}$ and ${\hat{f}}_{1j}^{\left(i\right)}$ to form the new predictor ${\tilde{f}}_{j}^{\left(i\right)},i=1,...,n-1.$. A detailed discussion about the construction of ${\tilde{\Pi }}_{n}$ in the special case of an independent instantaneously random PBN is given in [29], and the construction carries on with no changes for the case of a general PBN. One can immediately notice that the reduction mapping reduces the complexity of the original PBN not only by 'deleting' one gene but also by not increasing the number of BNs that comprise the reduced PBN.

Here, we want to point out the difference between the reduction and the projection mappings. While the projection is based on the probability distribution of a single gene, the reduction mapping is defined using the probability distribution of the entire collection of states of the given PBN which allows for the optimization procedure given in [29]. In both cases though, there is no control over the changes in the dynamics/state transition diagrams Γ*_j_* or the gene dependencies digraphs *G_j_* of the BNs comprising the original PBN. Indeed, one can easily find examples of PBNs such that both the reduction and the projection mappings significantly change those graphical representations of the structure and the dynamics of the model. In addition, both mappings rely on knowledge about the steady/stationary state distribution of the original PBN. For example, if one considers the state transition diagrams of the four contexts of the PBN $A^{q,p,4}\left(V,F,C\right),V=\left\{x_{1},x_{2},x_{3}\right\}$ Fig. (**1**), and applies the reduction mapping ${\tilde{\Pi }}_{3}$ that removes the rightmost gene from the network, one gets a PBN ${\tilde{A}}^{q,p,2}$ that has only 2 contexts as shown on Fig. (**2**). Thus, some of the important structure associated with the contexts ${\mathrm{BN}}_{3}$ and ${\mathrm{BN}}_{4}$ is lost.

The state transition diagram Γ of a PBN represents the dynamics of the network and has been related to both cellular types [11] and cellular states [12, 13]. Thus, it is desirable not to introduce significant changes in the structure of the attractor cycles and the sizes of the basins of attraction of Γ when reducing the size of the model. This kind of considerations led to the development the of *Dynamics Induced Reduction* (DIRE) algorithm in [28].

DIRE performs reduction of a PBN by deleting genes from the network while maximally preserving the dynamics of the constituent BNs and keeping their number unchanged. The algorithm collapses the state transition diagram for each individual BN in a manner similar to the example preceding the definition of the reduction mapping. States in each BN are aggregated/merged together if they differ only in the expression value of the gene *d* that has been deleted from the network. Because after 'deletion' *d* becomes a latent variable, the states **s** and ${\tilde{\text{s}}}^{d}$ 'collapse' to a state 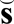 in the reduced network. The state 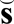 is obtained from either or ${\tilde{\text{s}}}^{d}$ by removing their *d* -th coordinate. During this procedure special attention has to be paid to both the *inconsistency points* in the state transition diagram, and to the states that become *absorbing* when the merging takes place. A state **s** in a BN is called an **inconsistency point** with respect to gene **d** if and only if its flipped state ${\tilde{\text{s}}}^{d}$ belongs to a different basin of attraction than the basin of attraction **s** belongs to. The one state **s** or ${\tilde{\text{s}}}^{d}$ that determines the position of the reduced state 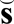 in the state transition diagram of the reduced BN is called **absorbing**.

The importance of the notion of inconsistency point is illustrated by examining the state transition diagram of the first context *BN*_1_ of the PBN depicted on Fig. (**1**).

Suppose that the gene *d* corresponding to the rightmost digit in the binary representation of the states is to be deleted. If one tries to collapse the state transition diagram, one should notice that, with respect to the attractor structure of the original BN, merging the leaf node (001) and the attractor node (000) can be done in two very different ways: either the merging happens towards the attractor state or it happens towards the leaf state. In the first case, the attractor state is preserved in the reduced BN as (00), and the basin of attraction of the attractor (111) in the original network looses one leaf. In the second case, the attractor structure of the reduced network differs significantly from that of the original BN, the only remaining attractor being the reduced state (11). Thus, if one considers the attractor structure of a BN as a representation of important biological characteristics of the real genomic regulatory system, then the merging of the states (000) and (001) should be done towards the attractor state. Notice that those two states are the only states in the original state transition diagram that create a possibility of essentially altering the attractor structure of the original BN. The rest of the states will merge within the basin of attraction of the attractor state (111). Thus, one possible set of absorbing states in this example is the set {(000),(101),(111),(011)}.

DIRE has several advantages compared to the reduction and the projection mappings. First, it optimally preserves the dynamical structure of the original PBN by controlling the damage to the attractor structure of its constituent BNs, by not increasing the maximum number of transitions required for states in a given basin of attraction to reach their attractor, and by preserving the number of the BNs that form the PBN, Fig. (**3**). Not only the attractor structure of the constituent BNs is preserved but there are no spurious attractors being generated in the reduced network. This is not the case for the projection and reduction mappings, as examples from [28] show. Second, the reduction mapping induced by the algorithm does not introduce changes in the number of the attractors nor in the length of the attractor cycles, unless there are points of inconsistency **s** that together with their flipped with respect to the 'deleted' gene *d* states ${\tilde{\text{s}}}^{d}$ are also attractor states as well. In addition, DIRE ensures that there will be a very little change, on average, of the relative sizes of the basins of the attraction. All of this together with the observation that the number *r* of the constituent BNs, their selection probabilities, and the parameters *p* and *q* remain the same for the original and the reduced PBN implies that, with the exception of the degenerate cases where there are relatively large number of attractor states that are points of inconsistency and whose flipped with respect to the 'deleted' gene *d* states are also attractor states, the steady-state distribution of the reduced PBN produced by the mapping will match closely the steady-state distribution of the original PBN. Moreover, the new algorithm does not require any prior information for the steady-state distribution of the original PBN as in the case of the projection and the reduction mappings. One can find a study of the DIRE performance on synthetically generated data: constrained PBN generated using the algorithm proposed in [30] and randomly generated PBN, on the complementary web site http://gsp.tamu.edu/Publications/dire.htm. The algorithm was also applied to a PBN model of a real gene regulatory network inferred from 31 malignant melanoma samples [17]. The 7-gene PBN $A^{0.01,0.01,4}$ with four contexts was built using the genes WNT5A, Pirin, S100P, RET1, MART1, HADHB, and STC2 [28]. The network model was reduced by "deleting" WNT5A using both the reduction mapping defined in Sec. III and DIRE algorithm. The steady-state distributions of the reduced networks were compared to the probability distribution P^*^ resulting from the collapsing procedure, Sec. III, which treats WNT5A as a latent variable. That comparison is shown on Fig. (**4**). The MSE differences between the steady-state distributions of the two reduced networks and P^*^ confirm that by controlling the damage on the state transition diagrams of the PBN's contexts DIRE can produce networks with steady-state distributions very similar to the original one [28]. Given the important biological interpretations of the steady-state distribution of the PBN model, DIRE reduction produces smaller and less complex networks which can be used to model the same phenomena as the larger ones.

Finally, the notion of point of inconsistency creates the opportunity for evaluating the importance of a particular gene for the network in question. This could lead to new methods for gene ranking, as well as options for applying control-based optimization which produces a favorable shift in the steady-state distribution of a given PBN and thus, could impact the design of therapeutic interventions.

## 4. THE PROBLEMS OF REDUCTION, INFERENCE AND CONTROL

The examples of reduction mappings in the previous section show that one should take into consideration both the dependencies among the genes in a GRN and the networks dynamics when designing reduction mappings. Thus, 'good' reduction mappings should preserve as much as possible the biologically meaningful properties of the original network model while taking care of its two major components: the digraph representing the dependencies among the genes and the set of functional relations that determine how the expression profile of each gene is predicted by the expression profiles of other genes. In this section we consider reduction mappings based on 'deletion' of one gene at a time from the network. We state the general reduction problem for PBNs and discuss different types of constraints. We also compare that general reduction problem to the problems of inference of the PBN model from data and to the problem of optimal control.

### 4.1. The Reduction Problem

Because every PBN is a collection of individual BNs endowed with a probability structure and gene mutation probability, we focus on reduction mappings for Boolean networks. Consider the space M*_n_* of all BNs on *n* genes. Then, having in mind the example of the multivalued projection mapping from the previous section, a reduction mapping can be defined as any set valued mapping $\Pi :{\text{M}}_{n}\to 2^{{\text{M}}_{n-1}}$ , the set of all subsets of Boolean networks on *n*-1 genes. Such a general definition takes into account only the 'deletion' of one of the genes from the networks in M*_n_*, and is of little practical use. On the other hand side, it helps to formulate the following

#### Reduction Problem

Given a set of constraints Λ and a BN $B\in {\text{M}}_{n}$ find a reduction mapping $\Pi :{\text{M}}_{n}\to 2^{{\text{M}}_{n-1}}$, such that every $\tilde{B}\in \Pi \left(B\right)$ satisfies Λ.

It is important to point out that the constraints Λ could be internal with respect to the model, i.e. related to the dynamical or static structure of $\tilde{B}$ (the graphs $\tilde{\Gamma }$ or $\tilde{G}$) or *B* (the graphs Γ or *G*), or external. For example, Λ could be related to qualitative knowledge/description of the biological phenomena being modeled.

Several observations are worth mentioning:

- Given a set of constraints Λ and a BN $B\in M_{n}$ the problem of finding 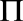 can be interpreted as a constrained search problem where the search space is the direct product $\times_{i=1}^{n-1}T_{i}$ of truth tables *T_i_* for the Boolean functions on *n* -1 variables. The set of constraints Λ determines some of the entries in those truth tables which could significantly reduce the size of the search space. This interpretation of the reduction problem allows for algorithms that are used to infer BNs from data, e.g. [30], to be used in determining the set $\Pi \left(B\right).$
- Given a set of constraints Λ and a reduction mapping 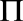 there exits a maximal, with respect to the partial order induced by set inclusion, subset $\Omega_{\Lambda ,\Pi }\subseteq {\text{M}}_{n}$ such that the same reduction mapping 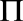 solves the reduction problem for every ${B\in \Omega }_{\Lambda ,\Pi },$ i.e. if ${B\in \Omega }_{\Lambda ,\Pi }$ then every $\tilde{B}\in \Pi \left(B\right)$ satisfies Λ.
- There is a partial order induced by set inclusion for the sets of constraints. If 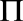 is a solution to the reduction problem for a given Λ_1_ and ${B\in \text{M}}_{n}$ , then 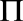 might not be a solution to the reduction problem for Λ_2_ and *B* if $\Lambda_{1}\subset \Lambda_{2}$. Thus, one can look for the maximal, with respect to this partial order, set of constraints Λ, $\Lambda_{1}\subset \Lambda$ so that 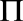 solves the reduction problem for Λ and *B*.
- Because one of the main reasons for constructing reduction mappings is to reduce the complexity of a network model, one can see that the choice of constraints Λ has a significant impact on achieving this goal. The cardinality of the set $\Pi \left(B\right)$ could be so big that the mapping 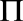 leads to an increase of the model complexity, as the example of the projection mapping shows. Moreover, the verification if a BN $\in \Pi \left(B\right)$ satisfies Λ can be computationally intensive for some sets of constraints. For example, if Λ = { a BN with singleton attractors only } then such a verification might require finding all of the attractor cycles in the state-transition diagrams Γ of the BNs $\in \Pi \left(B\right)$ - a problem known to be a NP-complete. The next example illustrates the trade-off between the size of $\in \Pi \left(B\right)$ and the cost to verify that all of the BNs $\in \Pi \left(B\right)$ satisfy the constraint Λ.

#### Example 1

The basic projection mapping ${\hat{\Pi }}_{n}$ is a solution of the reduction problem for the set of constraints $\Lambda =\left\{{\tilde{f}}^{\left(i\right)}={\hat{f}}_{0}^{\left(i\right)}\mathrm{or}{\hat{f}}_{1}^{\left(i\right)},i=1,...,n-1\right\}$ , where **f** is the network function for the BN that is being reduced. As mentioned earlier, the cardinality of the set ${\hat{\Pi }}_{n}$ (*B*) could be very large which leads to an increase of the model complexity. On the other hand side, the projection mapping has some advantages. First, because Λ prescribes all of the entries in the truth table of each $\tilde{B}$ in terms of the predictor functions for B, $\Omega_{\Lambda ,\Pi_{n}}\equiv {\text{M}}_{n}$ , i.e. the projection can be applied to every BN on *n* genes. Second, for the same reason, there is no need to verify that every BN $\in {\hat{\Pi }}_{n}\left(B\right)$ satisfies the constraint.

Next, we focus on establishing a minimal set of constraints based on the interpretation of the 'deleted' gene as a latent variable. The first constraint rises naturally from the local properties of the predictor functions and is based on the following Given a state s∈Γ, , the predictor function $f^{i}$ is called (**s**, *j*) independent if and only if it has partial derivative $\frac{\partial f^{i}}{\partial x_{j}}\left(\text{s}\right)=0$.

The (**s**, *j*) independence is a local property of the predictor function $f^{i}$ and suggests that if a toggle of $x_{j}$ in state **s** does not affect the prediction of gene $x_{i}$ then the new predictor function ${\tilde{f}}^{i}$ in any of the reduced networks $\tilde{B}$ should have the same value as $f^{i}$ (**s**) at the state 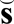 that is obtained from **s** by 'deleting' the *j* -th gene. Thus, we arrive at the local constraint Λ_1_ := { for every (**s**, *j*) independent 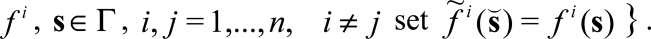 .

The second constraint Λ_2_ is also related to a local property but this time it is about the digraph of gene dependencies *G* . If one interprets a 'deleted' gene $x_{j}$ as a latent variable, then any two edges $x_{k}\to x_{j}$ and $x_{j}\to x_{i}$ in G should produce an edge $x_{k}\to x_{i}$ in $\tilde{G}$ for any of the reduced networks $\tilde{B}$. Thus, if the *j* -th gene is 'deleted' $\Lambda_{2}:=\left\{\text{ for every }x_{k}\to x_{j}\text{ and }x_{j}\to x_{i}\text{ set }{\tilde{W}}_{i}=\left\{W_{i}\backslash \left\{x_{j}\right\}\right\}\cup \left\{x_{k}\right\}\right\}$

One can combine Λ_1_ and Λ_2_ into a new constraint $\Lambda_{1}\cup \Lambda_{2}$ and then search for a solution to the reduction problem with respect to this constraint. The following example shows that although $\Lambda_{1}\cup \Lambda_{2}$ is biologically meaningful, it has little to do with the global dynamical properties of the state transition diagram Γ of *B*

#### Example 2

Consider the reduction problem for the constraint $\Lambda_{1}\cup \Lambda_{2}$ and the Boolean network $B\in {\text{M}}_{3}$ described by the truth table below:

**Table:** 

*x*_1_*x*_2_*x*_3_	*f*^1^	*f*^2^	*f*^3^
000	0	0	0
001	1	1	0
010	1	1	0
011	1	0	1
100	0	1	0
101	1	1	1
110	1	0	1
111	1	1	1

One can easily check that there are 10 networks in the set $\Pi \left(B\right)$ and all of those networks satisfy $\Lambda_{1}\cup \Lambda_{2}$ . At the same time, while the state-transition diagram of *B* has two singleton attractors, only one of those 10 networks has such a property. Moreover, 7 of them posses non-singleton attractors which shows that they cannot be used as reductions of the original BN in one wants to preserve the attractor structure of the network.

This example points out to the importance of constraints related to the global properties of Γ. At the same time, one has to be careful using properties of Γ as constraints when solving the reduction problem. For example, DIRE algorithm uses the the entire state-transition diagram as a constraint but applying it to the network *B* from the above example produces $\tilde{B}$ that does not possess the dependency $x_{2}\to x_{2}$ that is present in the gene dependencies digraph *G* of *B* . Thus, in some cases, the entire state-transition diagram appears to be too strong of a constraint when solving the reduction problem.

### 4.2. Reduction and Inference

The interpretation of the reduction problem as a search problem points out to its similarity to the problem about PBN inference from data. Constraints that are used when one designs network models from data could be also used when solving the reduction problem. For example, it was shown that genes which predict each other have a profound effect on the attractor structure of a BN [31]. The next definition specifies the notion of such gene interdependencies. The genes $x_{i}$ and $x_{i}$ in a BN are said to have a **bidirectional relationship** if and only if $x_{i}\in W_{j}$ and $x_{j}\in W_{i}$. The relationship is said to be of **connectivity n** if Wi=Wj=n.

Bidirectional relationships are important when the inference from data is restricted to the subclass $B_{s}\subset {\text{M}}_{n}$ of Boolean networks with singleton attractors only. The subclass ${\text{B}}_{s}$ is important in situations where inference is made from time-independent data - the kind of data one usually gets in microarray studies involving human subjects. It is common to assume that in those cases data come from the steady-state of the genomic regulatory system which in its turn implies that the majority of data points represent attractors of the modeled system. The attractor cycles in BNs that model biological networks are typically associated with phenotypes and tend to be short [11], with biological state stability contributing to singleton attractors [32]. The results presented in [31] show that bidirectional gene relationships in a Boolean network are a common cause for the presence of non-singleton attractors in its state-transition diagram. One might guess that the creation of bidirectional relationships by the reduction mapping under the given set of constraints contributes to the spurious attractor cycles in most of the networks from $\Pi \left(B\right)$ . This points out to the importance of tricycles $x_{i}\to x_{j}\to x_{k}\to x_{i}$ in the digraphs *G* of BNs $\in B_{s}$, and leads to a new constraint Λ_3_ for the reduction problem for the subclass ${\text{B}}_{s}$ . The reduction problem under the constraint Λ_3_ = {*the ´deleted´ gene does not participate in any tricycle in the original G*} was considered in [33]. The paper presents an algorithm that solves the reduction problem for the set of constraints $\Lambda_{1}\cup \Lambda_{3}$ . The mapping 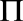 produced by the algorithm does not ensure that for a $B\in {\text{B}}_{s}$ all of the reduced networks in $\Pi \left(B\right)$ have singleton attractors only. However, the probability of recovering the original attractor structure of a Boolean network with singleton attractors only is much higher when the constraint Λ_3_ is used in conjunction with Λ_1_ compared to the case when only Λ_1_ is used as a constraint.

The similarity between the problems of reduction and inference of PBNs could be explored in the other possible direction: using the knowledge about reduction mappings to infer the model from data. Constraints used by the DIRE algorithm for reducing BNs could be applied during an inference procedure [34]. Because attractor cycles are processed independently of their basins and special care is taken when a point of inconsistency is encounter during reduction one can use DIRE as a constraint in designing PBNs from data, especially when data are believed to come from the steady-state of the underlying genomic regulatory system. DIRE mapping induces a partial order in every subset of data points that is considered to be part of set of fixed points of the regulatory system. The algorithm given in [34] uses this partial order to infer a PBN from data so that the model's attractor structure is stable with respect to the DIRE reduction. A real data example using a melanoma data set [17], could be found on *http://gsp.tamu.edu/Publications/ BNs/dire_ranking.pdf*.

### 4.3. Reduction and Control

Probabilistic Boolean networks have been the model of choice in studying optimal regulatory intervention. The reason for that lies in the well developed theory of Markov chains and the associated transition probability matrices. To address the issue of changing the long-run behavior, stochastic control has been employed to find stationary control policies that affect the steady-state distribution of a PBN. The algorithms used to find these solutions have complexity which increases exponentially with the number of the genes in the network. Hence, there is a need for size-reducing mappings producing new and more tractable models whose stationary control policies induce sub-optimal stationary control policies on the larger PBN. This subsection focuses on a specific stationary control policy, Mean-First-Passage-Time control policy, and reviews the two major issues that link the reduction problem to the problem about designing the MFPT control policy. The first problem concerns the effects of the reduction mappings introduced in [29] on that policy. The second issue is about how MFPT control policies that are designed on the reduced network can be extended to stationary control policies on the original network. We concentrate on Boolean networks with a perturbation *BN*_*p*_ - the building blocks of a PBN.

The type of reduction policy introduced in [29] rests on the following procedure. If one assumes that the gene *d* is going to be 'deleted' from the given *BN*_p_ then for every pair of states **s** and ${\tilde{\text{s}}}^{d}$ in the state space *S* , one can consider the states **w** and **v** for which **s** → **w** and ${\tilde{\text{s}}}^{d}$ → **v**. Because after 'deletion' becomes a latent variable, the states **s** and ${\tilde{\text{s}}}^{d}$ 'collapse' to a state 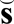 in the reduced network. The state 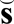 is obtained from either **s** or ${\tilde{\text{s}}}^{d}$ by removing their *d* -th coordinate. The reduction mapping, denoted by ${\tilde{\Pi }}_{d}$ , constructs the truth table of the reduced network by selecting the transition 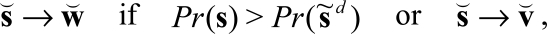 otherwise. This particular type of reduction is a special case of a reduction mapping $\Pi_{v^{d}}$ induced by a selection policy $v^{d}$ which is defined next [35]. A selection policy $v^{d}$ corresponding to the 'deleted' gene *d* is a $2^{n}$ dimensional vector $v^{d}\in {\left\{0,1\right\}}^{2^{n}}$, indexed by the states of *S* and having components equal to 1 at only one of the positions corresponding to each pair $\left(\text{s},{\tilde{\text{s}}}^{d}\right),\text{s}\in S$ .

Notice that for each gene *d* there are $2^{2^{n-1}}$ different selection policies corresponding to that gene. Using this definition, one can define a general $v^{d}$of a reduction mapping -induced reduction mapping $\Pi_{v^{d}}$ . The mapping constructs the truth table of the reduced network by selecting the transition 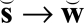 if 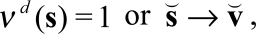, otherwise. To each selection policy $v^{d}$ there corresponds a matrix ${\text{F}}_{v^{d}}$ which is called the *companion matrix* for the reduction mapping $\Pi_{v^{d}}$ [35]. The fundamental matrix can be easily obtained from the transition probability matrix of the given *BN_p_* . Moreover, the transition probability matrix 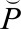 of the reduced network $\Pi_{v^{d}}$ *BN_p_* has entries that can be computed, e.g. sec. 2, and can be shown to be identical (up to a very small perturbation) to ${\text{F}}_{v^{d}}$ . Thus one can study the effects of selection policy-induced reduction mappings on both the MFPT control policy designed for the reduced network and on the similarity between the steady-state distributions of the original and the reduced networks. Following the notation introduced in sec. 2 we consider the the MFPT stationary control policy $\pi_{g,\gamma }=\left\{\times_{g,\gamma },\times_{g,\gamma },...\right\},$ designed using the MFPT algorithm from [23] for the *BN_p_* with γ as a parameter and *g* being the control gene. Because 'deleting' of the gene *d* has to be interpreted as a creation of a latent or non-observable variable, it is desirable that the MFPT control policy 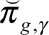 with the same parameter γ for the reduced network 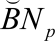 is as close as possible to the one designed for the original network. In this way, one can achieve similar control actions for every state s,s∈S and its corresponding reduced state 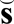. Taking advantage of the properties of the companion matrix ${\text{F}}_{v^{d}}$ for the mapping $\Pi_{v^{d}}$ one can show that there exists a selection policy $v_{{}^{\circ}}^{d}$ that minimizes the relative effect on the stationary MFPT control policy $\pi_{g,\gamma }$ designed for *BN_p_* among the all of possible selection policies *v*^*d*^ [35]. The relative effect on the policy $\pi_{g,\gamma }$ is measured by comparing the number of times where the control for the reduced states 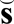 differs from the control $\pi_{g,\gamma }$ **(s), s** is such that ***v^d^*(s)=1**, to the number of times where there is no change in the control action.

The problem of inducing control on the larger network model using a control policy designed on the reduced model is an example of an ill-posed inverse problem, and has not been studied extensively. The MFPT control policy 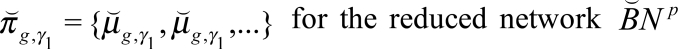 has a dimension of $2^{n-1}$ while all of the stationary control policies for the original network *BN_p_* are of dimension of $2^{n}$ . Recently, Ghaffari *et al*. [36] considered this inverse problem for the reduction mapping defined in [29]. That reduction mapping is induced by a selection policy that is determined by the steady-state distribution of the *BN_p_* that is being reduced. There are some obvious constraints on the possible ways to extend 
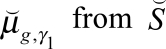
-the state space of the reduced network 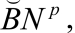 to *S* - the state space of the original network *BN_p_*. The definition of the reduction mapping 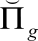 shows that to each state 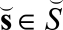 there are two corresponding states: **s** and its flipped state ${\tilde{s}}^{g}$ in *S* that differ only in the value of the gene *g* . Therefore, under the interpretation of the 'deleted' gene *g* as a latent variable we should have 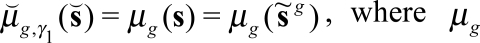 is the extension of 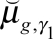. Thus, the goodness of extension of the control policy 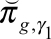 can be measured by the normalized Hamming distance between the vectors $\mu_{g}$ and $\mu_{g,\gamma }$ . The simulation study performed in [36] used randomly generated sets of *BN*^0.1^, on *n* = 7 genes. Each set shared a common set of attractor states with no restrictions on the way they formed attractor cycles. In addition, the cardinalities $\#W_{i}$ of the gene predictor sets were restricted to no be no larger than 3 to keep the networks from being chaotic. Furthermore, the two parameters *γ_1_* and *γ* were set equal because in practical applications one would often assume similar costs for the original and the reduced networks. The study showed that one can expect relatively significant differences for the two control policies only for very small values of *γ* , Fig. (**5**). For *γ* > 2 the MFPT control policy on the original networks differed less than 3% from the stationary control policy that was induced by the MFPT control policy on the reduced networks. Thus, one can use the reduced network to accurately estimate the MFPT control policy for a large interval of the parameter *γ* that is associated with a relative high cost of control. The difference in the average behavior of the two sets of networks presented on Fig. (**5**) suggests that the attractor structure of the models plays an important role in solving this inverse problem.

## 5. CONCLUSIONS

High complexity is a major impediment when computational models of genomic regulation are used to design optimal strategies for therapeutic intervention and control of disease. Thus, mappings which reduce the size/complexity of the model while preserving its important structural and dynamical characteristics become indispensable for its successful applications. The reduction problem in its very general formulation emphasizes the role of constraints in the process of designing reduction mappings for probabilistic Boolean networks. It also provides the basis for the comparison drawn between the problems of reduction and inference of PBNs from gene expression data. The similarity between the two problems allows for using reduction mappings in the process of network inference, and the application of known inference algorithms in designing reduction mappings. There is also a similarity between the problems of reduction and control when MFPT stationary control policy and selection policy-induced reduction mappings are considered. This provides the basis for investigating the question about the effects of reduction mappings on the MFPT control policy for the reduced network, and also the question of the possibility to use the MFPT control policy for the reduced network to induce a stationary control policy for the original PBN that approximates its original MFPT control policy. To date, very little is known about the cost of applying reduction mappings. A result of preliminary nature is presented in [37] where for the first time the stochastic complexity was used for that purpose. Estimating the reduction cost is important because it is not advantageous to produce less complex model by a highly complex/expensive mapping. Furthermore, little is known about estimates of the computational savings when a reduced version of a large network is used. Clearly, the reduced models have their state space exponentially smaller compare to those of the larger PBNs. Carefully designed large scale simulation studies could provide hints about how the computational burden of using large network models of genomic regulation compares to their reduced under different sets of constraints versions.

## Figures and Tables

**Fig. (1):**
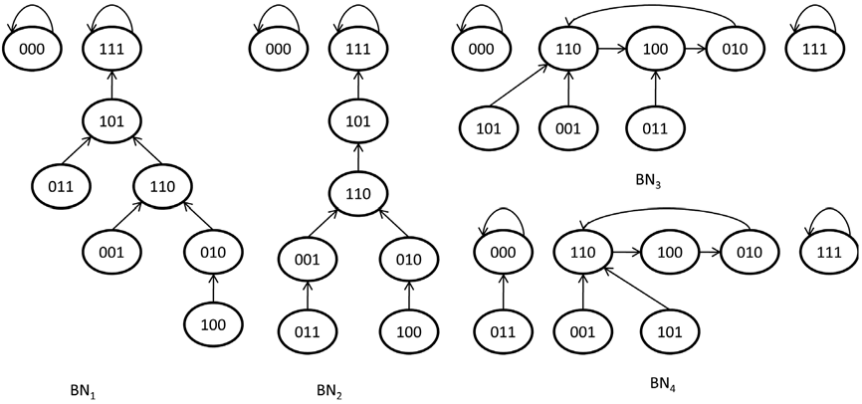
State transition diagrams of the four contexts of the PBN *A*^*q, p*,4^ (*V, F,C*) .

**Fig. (2):**
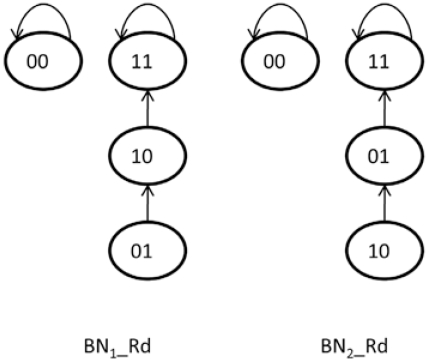
State transition diagrams of the two contexts of the reduced model ${\tilde{A}}^{q,p,2}$ obtained after applying the reduction mapping with respect to the third gene to the PBN presented on Fig. (1).

**Fig. (3):**
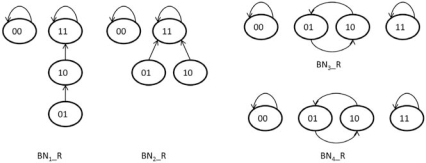
State transition diagrams of the contexts of the reduced model obtained after applying DIRE with respect to the third gene to the PBN presented on Fig. (1).

**Fig. (4):**
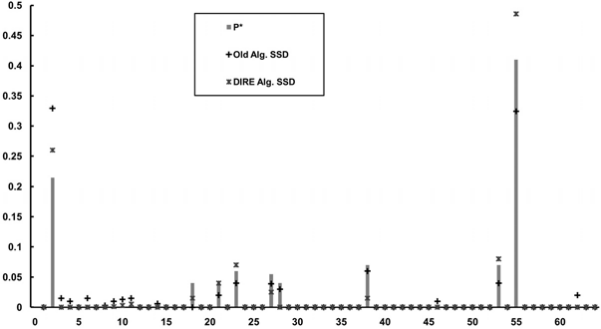
Steady-state distributions for the reduced networks that were obtained by applying the reduction algorithm and DIRE on the 7-gene melanoma related PBN.

**Fig. (5):**
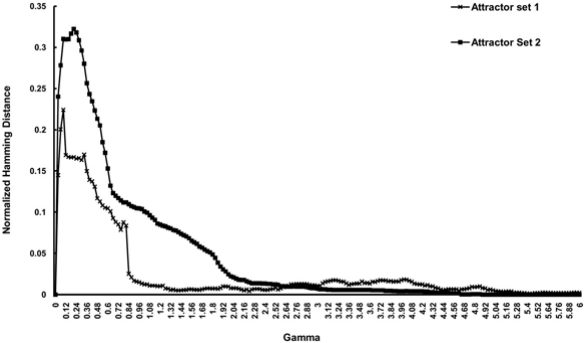
Average effects of reduction on the control policy.

## References

[1] Dougherty ER, Braga-Neto U (2006). Epistemology of computational biology: mathematical models and experimental prediction as the basis of their validity. J. Comput. Biol.

[2] de Jong H (2002). Modeling and Simulation of Genetic Regulatory Systems: A Literature Review. J. Comp. Biol.

[3] Crick F (1970). Central dogma of molecular biology. Nature.

[4] Ivanov I, Dougherty ER (2006). Modeling Genetic Regulatory Networks: Continuous or Discrete?. Biol. Syst.

[5] Kauffman S A (1969). Metabolic Stability and Epigenesis in Randomly Constructed Genetic Nets. J. Theor. Biol.

[6] Pal R, Datta A, Fornace AJ, Bittner ML, Dougherty ER (2005). Boolean relationships among genes responsive to ionizing radiation in the NCI 60 ACDS. Bioinformatics.

[7] Shmulevich I, Dougherty ER, Kim S, Zhang W (2002). Probabilistic Boolean Networks: a rule-based uncertainty model for gene regulatory networks. Bioinformatics.

[8] Brun M, Dougherty ER, Shmulevich I (2005). Steady-State Probabilities for Attractors in Probabilistic Boolean Networks. Signal Processing.

[9] Kauffman S A (1969). Homeostasis and differentiation in random genetic control networks. Nature.

[10] Shmulevich I, Dougherty ER, Zhang W (2002). Gene perturbation and intervention in Probabilistic Boolean Networks. Bioinformatics.

[11] Kauffman SA (1993). The Origins of Order: Self-Organization and Selection in Evolution.

[12] Huang S (1999). Gene expression profiling, genetic networks, and cellular states: an integrating concept for tumorigenesis and drug discovery. J. Mol. Med.

[13] Huang S (2001). Genomics, complexity and drug discovery: Insights from Boolean network models of cellular regulation. Pharmacomgenomics.

[14] Shmulevich I, Dougherty ER, Zhang W (2002). From boolean to probabilistic boolean networks as models of genetic regulatory networks. Proc. IEEE.

[15] Shmulevich I, Lahdesmaki H, Dougherty ER, Astola J, Zhang W (2003). The role of certain Post classes in Boolean network models of genetic networks. PNAS.

[16] Faryabi B, Chamberland JF, Vahedi G, Datta A, Dougherty ER (2008). Optimal Intervention in Asynchronous Genetic Regulatory Networks. IEEE J. Selected Top. Signal Proc.

[17] Bittner M, Meltzer P, Chen Y, Jiang Y, Seftor E, Hendrix M, Radmacher M, Simon R, Yakhini Z, Ben-Dor A, Sampas N, Dougherty E R, Wang E L, Marincola F, Gooden C, Lueders J, Glatfelter A, Pollock P, Carpten J, Gillanders E, Leja D, Dietrich K, Beaudry C, Berens M, Alberts D, Sondak V, Hayward N, Trent J (2000). Molecular classification of cutaneous malignant melanoma by gene expression profiling. Nature.

[18] Datta A, Pal R, Choudhary A, Dougherty ER (2007). Control Approaches for probabilistic gene regulatory networks. IEEE Signal Proc. Mag.

[19] Datta A, Choudhary A, Bittner M, Dougherty ER (2003). External control in markovian genetic regulatory networks. Mach. Learn.

[20] Datta A, Pal R, Dougherty ER (2006). Intervention in probabilistic gene regulatory networks. Curr. Bioinformat.

[21] Pal R, Datta A, Dougherty ER (2006). Optimal infinite-horizon control for Probabilistic Boolean Networks. IEEE Trans. Signal Processing.

[22] Akutsu T, Hayashida M, Ching W-K, Ng MK (2007). Control of Boolean networks: Hardness results and algorithms for the tree structured nqtworks. J. Theor. Biol.

[23] Vahedi G, Faryabi B, Chamberland J-F, Data A, Dougherty ER (2008). Intervention in gene regulatory networks via a stationary Mean-first-Passage-Time control policy. IEEE Trans. Biomed. Eng.

[24] Shmulevich I, Dougherty ER, Zhang W (2002). Control of stationary behavior in probabilistic Boolean networks by means of structural intervention. Biol. Syst.

[25] Norris J (1998). Markov Chains.

[26] Bertsekas DP (2005). Dynamic Programming and Optimal Control.

[27] Vahedi G, Data A, Dougherty ER (2007). Which Control Gene Should be Used in Genetic Regulatory Networks. IEEE Statistical Signal Processing Workshop. Madison.

[28] Ivanov I, Pal R, Dougherty ER (2007). Dynamics preserving size reduction mappings for probabilistic Boolean networks. IEEE Trans. Signal Proc.

[29] Ivanov I, Dougherty ER (2004). Reduction mappings between Probabilistic Boolean Networks. EURASIP JASP.

[30] Pal R, Ivanov I, Datta A, Dougherty ER (2005). Generating Boolean Networks with a Prescribed Attractor Structure. Bioinformatics.

[31] Vahedi G, Ivanov I, Dougherty ER (2009). Inference of Boolean Networks under Constraint on Bidirectional Gene Relationships. IET Syst. Biol. J.

[32] Zhou XB, Wang XD, Pal R, Ivanov I, Bittner M, Dougherty ER (2004). A Bayesian connectivity-based approach to constructing probabilistic gene regulatory networks. Bioinformatics.

[33] Ivanov I, Vahedi G, Dougherty ER (2007). Constrained Reduction Mapping for a Class of Network Models of Genomic Regulation. Life Science Systems & Applications Workshop (LISA 2007).

[34] Ivanov I, Pal R, Dougherty ER (2006). Applying Reduction Mappings in Designing Genomic Regulatory Networks. Life Science Systems & Applications Workshop (LISA 2006).

[35] Ivanov I, Simeonov P, Ghaffari N, Qian X, Dougherty ER (2009). Selection Policy Induced Reduction Mappings for Boolean Networks. J. Theor. Biol.

[36] Ghaffari N, Ivanov I, Dougherty ER (2008). Reduction Mappings and Control Policies for Intervention in Boolean Networks. IEEE International Workshop on Genomic Signal Processing and Statistics (GENSIPS 2008).

[37] Dougherty J, Ivanov I (2008). Reduction Cost for Boolean Networks with Perturbation. IEEE International Workshop on Genomic Signal Processing and Statistics (GENSIPS 2008).

